# The complete mitochondrial genome of *Dascyllus trimaculatus* (Rüppell, 1829)

**DOI:** 10.1080/23802359.2022.2161838

**Published:** 2023-01-08

**Authors:** Juliana Limón, May B. Roberts, Darrin T. Schultz, Giacomo Bernardi

**Affiliations:** a Department of Ecology and Evolutionary Biology, University of California Santa Cruz, Santa Cruz, CA, USA; b Department of Molecular Evolution and Development, University of Vienna, Vienna, Austria; c Monterey Bay Aquarium Research Institute, Moss Landing, CA, USA; d Department of Biomolecular Engineering and Bioinformatics, University of California, Santa Cruz, CA, USA

**Keywords:** *Dascyllus trimaculatus* mitochondrial genome, Pomacentridae

## Abstract

Damselfishes (family Pomacentridae) comprise approximately 400 species that play an important ecological role in temperate and coral reefs. Here, for the first time, we assemble and annotate the mitochondrial genome of *Dascyllus trimaculatus*, the three-spot dascyllus, a planktivorous damselfish that primarily recruits in anemones. The circular genome of *D. trimaculatus* is 16,967 bp in length and contains 13 protein-coding genes, 22 transfer RNA genes, two ribosomal RNA genes, and a control region. Gene arrangement and codon usage is similar to reported mitochondrial genomes of other damselfish genera, and a phylogenetic analysis of a set of damselfish representatives is consistent with known evolutionary analyses.

The family Pomacentridae, commonly known as damselfishes, contains 29 genera and over 400 species (Allen 1991; Tang et al. 2021), with most of these species occurring in the Indo-west and central Pacific region (Allen 1991; Tang et al. 2021). The three-spot damselfish, *Dascyllus trimaculatus* has a wide distribution that extends from the Red Sea to the Central Pacific and belongs to a species complex with three other recognized species (Bernardi et al. 2001; Leray et al. 2010). Here, we present, for the first time, the fully assembled mitochondrial genome of *D. trimaculatus* (Rüppell, 1829) to aid in future studies of this species complex. We placed this genome in its evolutionary context within the Pomacentridae family, and confirmed the geographic origin of our specimen using a tree based on the control region (d-loop) of several other *D. trimaculatus.*

A juvenile (38 mm TL) *Dascyllus trimaculatus* was acquired through the aquarium trade (LiveAquaria.com), and euthanized following an approved IACUC protocol animal use (Berng 1701.r2). The specimen was deposited at the University of California Museum of Natural History under the voucher number DTR_Kuro_0920G (mabrober@ucsc.edu). Total genomic DNA was extracted using muscle tissue from the individual (Qiagen DNeasy Blood & Tissue Kit, Hilden, Germany). A shotgun/whole genome library was prepared and sequenced using a MiSeq Illumina sequencing platform in the Paleogenomics Laboratory at the University of California Santa Cruz resulting in 2,356,971 contigs of 150 bp length each. Data were aligned with the closely related *Abudefduf vaigiensis* (GenBank: NC_009064) in Geneious R11 and trimmed to create a consensus sequence (Geneious, Auckland, New Zealand, https://www.geneious.com). The final genome was then annotated using MitoAnnotator (Iwasaki et al. 2013). The mitochondrial genome of *D. trimaculatus* was found to be 16,967 bp in length and contains 13 protein-coding genes, 22 transfer RNA genes, two ribosomal RNA genes, and a control region (SRA: SRR20942050, Run: SRR20942050, BioProject: PRJNA828170, BioSample: SAMN27642109-DTR_Kuro_0920G; GenBank sequence accession number ON556619) (Supplementary Figure S1). The genome has an overall base composition of 28.54% A, 29.41% C, 15.64% G, and 26.42% T. This composition is similar to the mitogenomes of other vertebrates, with a higher AT (54.95%) than GC (45.05%) content. A comparison of mutations (after removing the variable control region) between *D. trimaculatus* and its close relative *Chromis viridis* (see below) showed, as expected, a higher rate of transitions (10.31%; *R* = 3.17%, *Y* = 7.14%) than transversions (4.62%; *M* = 2.13%, *W* = 1.42%, *S* = 0.63%, *K* = 0.43%) resulting in a tr/tv ratio of 2.3.

A Neighbor-Joining tree based on mitochondrial genomes (all regions except the variable control region, i.e. an alignment of 16,178bp) of seven damselfish species (*Dascyllus trimaculatus*, *Chromis notata*, *Chromis viridis*, *Abudefduf vaigiensis*, *Amblyglyphidodon curacao*, *Chrysiptera biocellata*, and *Amphiprion ocellaris*) and one surfperch, Embiotocidae (*Embiotoca jacksoni*) to serve as an outgroup (following Tang et al. 2021) was established using PAUP (Swofford 2003). Node confidence was obtained by running 1000 bootstrap replicates (Felsenstein 1985; Figure 1).

**Figure 1.**
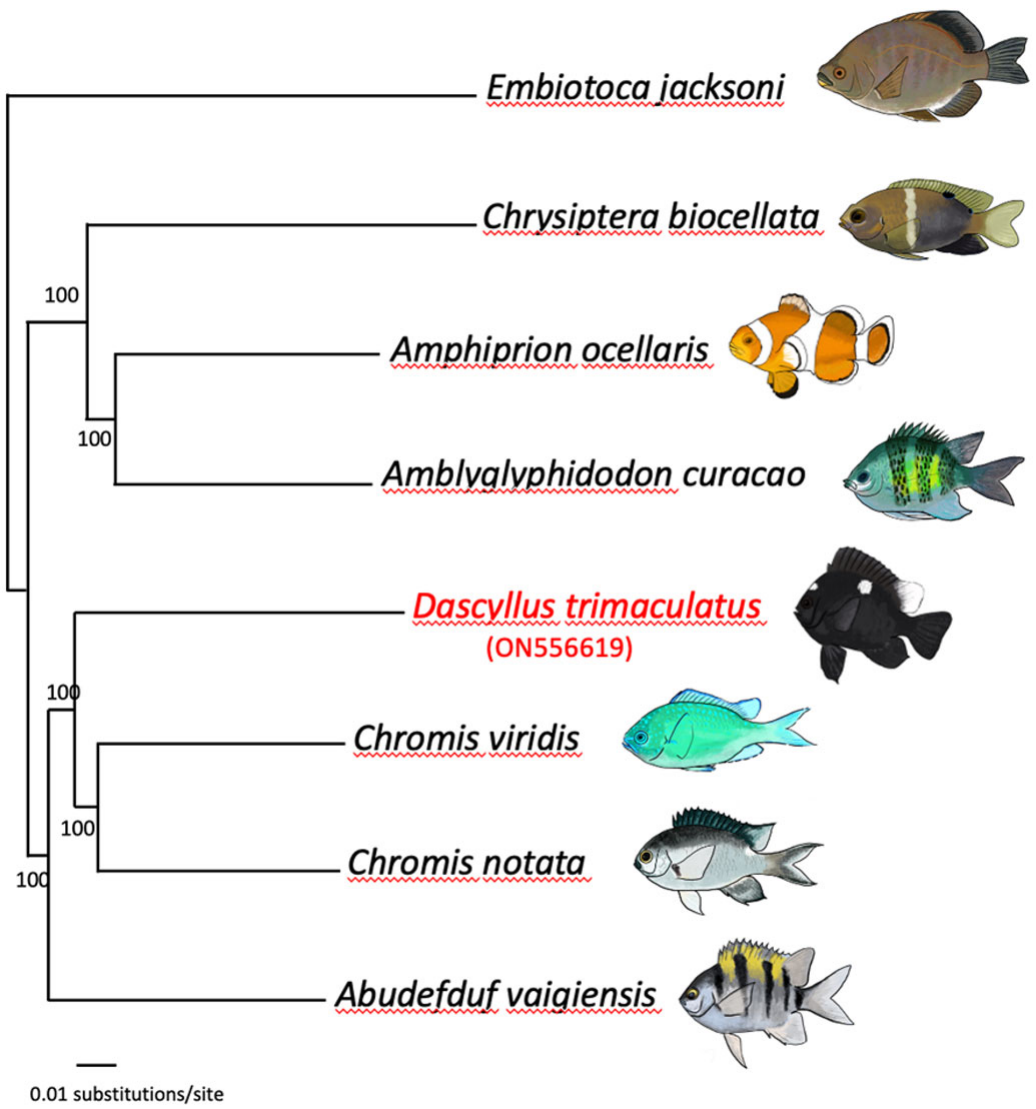
Phylogenetic relationships based on complete mitochondrial genomes of representative damselfish species and one surfperch outgroup (Black Surfperch, *Embiotoca jacksoni*). Numbers at nodes represent bootstrap values. The focal species of this study, *Dascyllus trimaculatus*, is labeled in red font. GenBank accession numbers are indicated between parentheses under the species names: *Embiotoca jacksoni* (NC_029362), *Chrysiptera biocellata* (NC_040304), *Amphiprion ocellaris* (NC_009065), *Amblyglyphidodon curacao* (NC_043918), *Dascyllus trimaculatus* (ON556619), *Chromis viridis* (MT199208), *Chromis notata* (NC_052722), and *Abudefduf vaigiensis* (NC_009064). Original drawings by MBR.

In addition, a Neighbor-Joining phylogenetic tree based on 375 bp of the variable control region (D-loop) of our sample and an additional 636 different *D. trimaculatus* individuals (obtained from the literature, Leray et al. 2010) was established to locate the geographic origin of our sample (Supplementary Figure S2). Results suggests that the organism we sequenced is from the West Pacific Rim clade, with closest individuals from the Visayas (Philippines) and Manado (Indonesia).

## Data Availability

The genome sequence data that support the findings of this study are openly available in GenBank of NCBI at https://www.ncbi.nlm.nih.gov under the accession no. ON556619. The associated BioProject, SRA, and Bio-Sample numbers are PRJNA828170, SRR20942050, and SAMN27642109-DTR_Kuro_0920G, respectively.
